# Differential Expression of Matrix Metalloproteases in Human Fibroblasts with Different Origins

**DOI:** 10.1155/2012/875742

**Published:** 2012-03-04

**Authors:** Diana Lindner, Christin Zietsch, P. Moritz Becher, Karsten Schulze, Heinz-Peter Schultheiss, Carsten Tschöpe, Dirk Westermann

**Affiliations:** Department of Cardiology and Pneumology, Charité Universitätsmedizin Berlin, Campus Benjamin Franklin, Hindenburgdamm 30, 12200 Berlin, Germany

## Abstract

Fibroblasts are widely distributed cells and are responsible for the deposition of extracellular matrix (ECM) components but also secrete ECM-degrading matrix metalloproteases. A finely balanced equilibrium between deposition and degradation of ECM is essential for structural integrity of tissues. In the past, fibroblasts have typically been understood as a uniform cell population with comparable functions regardless of their origin. Here, we determined growth curves of fibroblasts derived from heart, skin, and lung and clearly show the lowest proliferation rate for cardiac fibroblasts. Furthermore, we examined basal expression levels of collagen and different MMPs in these three types of fibroblasts and compared these concerning their site of origin. Interestingly, we found major differences in basal mRNA expression especially for MMP1 and MMP3. Moreover, we treated fibroblasts with TNF-*α* and observed different alterations under these proinflammatory conditions. In conclusion, fibroblasts show different properties in proliferation and MMP expression regarding their originated tissue.

## 1. Introduction

Fibroblasts are a heterogeneous population of cells found in numerous tissues and are of mesenchymal origin. The most common products secreted by fibroblasts are components of the extracellular matrix (ECM) such as different types of collagen, fibronectin, and proteoglycans [1–5]. The ECM preserves the geometry and structural integrity of various organs and tissues, but it is not only a scaffold that provides support for cells, but is further involved in cell-cell interactions, proliferation, and migration. The dermis is composed of the predominating ECM and the fewer cellular constituents of mainly fibroblasts which provides flexibility and physical strength to skin [6]. In the heart fibroblasts are 70% of the cells in the myocardium, whereas myocytes occupy two-thirds of the tissue volume [7–9]. Pulmonary fibroblasts are also present in the connective tissue of the airway wall under the surface epithelium [10].

Moreover, fibroblasts are not only involved in the deposition of ECM products but also secrete ECM-degrading enzymes called matrix metalloproteases (MMPs) and therefore are involved in the degradation of ECM. These MMPs are zinc-dependent endopeptidases secreted by fibroblasts and functioning extracellularly [2, 4, 5, 11]. Their substrates are marcomolecules of the ECM network consisting of different collagen types, proteoglycans, and glycoproteins. They are classified as collagenases, gelatinases, stromelysins, and membrane-type MMPs according to their substrate specificity. Collagenases cleave collagens at unique sites generating 75–25 kDa fragments called gelatins which were further degraded into single amino acids or oligopeptids by the gelatinases [12].

In this study we isolated three types of human fibroblasts, namely, cardiac, dermal, and pulmonary fibroblasts. We investigated the proliferation of fibroblasts in accordance to their different tissue origins to determine whether tissue-specific proliferation rates of this cell type can be found.

Furthermore, fibroblasts play a key role in normal matrix turnover as well as in pathological matrix deposition or degradation which can occur, for instance, during fibrosis or inflammation. We hypothesized tissue-specific expression levels of MMPs in the different types of fibroblasts. Therefore, we determined the basal MMP expression of primary human fibroblasts derived from heart, lung, and skin. Thereby we focused on these MMPs which are described to be expressed in cardiac tissue [2].

TNF-*α* is a proinflammatory cytokine, which plays a key role in myocardial inflammatory response after myocardial infarction [13]. In the early stage, the predominant sources of TNF-*α* are the infiltrated inflammatory cells. In this study we demonstrate different MMP expression of fibroblasts according to their site of origin in response to TNF-*α*.

## 2. Materials and Methods

### 2.1. Isolation and Culture of Human Cardiac, Dermal, and Pulmonary Fibroblasts

Human cardiac, dermal, and pulmonary fibroblasts were obtained by outgrowth from biopsies from four, three, or two patients, respectively. The endomyocardial biopsies were obtained from the right side of the ventricular septum of patients with dilated cardiomyopathy (ejection fraction < 30). Used was a flexible bioptome via the femoral vein approach. For dermal fibroblasts skin tissue which remained after surgery was used. The lung biopsies were obtained by bronchoscopy.

Primary human fibroblasts were cultured in Iscove's basal medium (Biochrom AG, Berlin, Germany) containing 10% human serum, 10% fetal calf serum, 100 U/mL penicillin, and 100 *μ*g/mL streptomycin (PAA, Cölbe, Germany) at 37°C in a humidified atmosphere with 5% CO_2_ and 95% air. All further experiments were done with cells between the second and the fourth passage.

### 2.2. Immunofluorescence Staining of Primary Human Fibroblasts

Cells to be characterized were seeded out into 48-well plates and grown to approximately 80% confluence. Cells were then fixed in 4% paraformaldehyde at room temperature for 10 minutes. Subsequently, cells were permeabilized with 0.25% Triton X-100 in TBS for 10 minutes followed by blocking with TBS containing 0.25% Triton X-100 and 1.5% normal rabbit serum for vimentin and desmin staining or 1.5% normal horse serum for CD31 and P4HB staining for 30 minutes at room temperature using the sera of Vectastain Elite ABC Kit and the avidin solution (Vector Laboratories, Burlingame, CA). Cells were exposed to a goat anti-vimentin antibody (Sc-7558, Santa Cruz, Heidelberg, Germany) or a goat anti-desmin antibody (Sc-7559, Santa Cruz, Heidelberg, Germany) in the presence of the biotin solution (Vector Laboratories, Burlingame, CA) for 90 minutes followed by 60-minute incubation with a biotinylated secondary anti-goat antibody raised in rabbit. For CD31 and P4HB visualization cells were incubated with an mouse anti-CD31 antibody (M0823, Dako, Hamburg, Germany) or a mouse anti-P4HB antibody (H00005034-B01P, Abnova, Heidelberg, Germany) in the presence of the biotin solution (Vector Laboratories, Burlingame, CA) for 90 minutes followed by incubation with a biotinylated secondary anti-mouse antibody raised in horse for 60 minutes. For fluorescence visualization cells were finally incubated with 9 *μ*g/mL Cy3-conjugated streptavidin (016-160-084, Jackson ImmunoResearch, Suffolk, UK) diluted in TBS containing 0.25% Triton X-100 for 30 minutes followed by a nuclear staining with Dapi (D1168, Invitrogen, Darmstadt, Germany) for 10 minutes. Cells were covered with TBS and analysed by fluorescence microscopy.

### 2.3. Proliferation of Primary Human Fibroblasts

To determine the doubling time of fibroblasts they were seeded out into 96-well plates and allowed to attach for 48 hours. Every second or third day only three wells of the 96-well plates were incubated with CellTiter-Blue viability solution (Promega, Mannheim, Germany) diluted in complete cell culture medium as recommended and incubated at 37°C in a humidified atmosphere with 5% CO_2_ and 95% air for 2 hours. The colour development was monitored at 560 nm with a reference wave length of 620 nm. Growth curves were plotted as x-fold of reference corrected optical density (OD) compared to the first measurement 48 hours after seeding. The doubling time was calculated using following formula, DT = [*t* − *t*_0_]log⁡2/[log⁡*N* − log⁡*N*_0_], where *N* and *N*_0_ are the detected OD_560–620_ at times *t* and *t*_0_, respectively.

### 2.4. Stimulation of Human Fibroblasts

For stimulation experiments the primary human fibroblasts were seeded out into 24-well plates. When the culture was about 80% confluence, cells were washed once and serum-starved in Iscove's basal medium (Biochrom AG, Berlin, Germany) containing 0.5% FCS, 100 U/mL penicillin, and 100 *μ*g/mL streptomycin (PAA, Cölbe, Germany) 16 hours prior to the experiment. Cells were then treated with a final concentration of 10 ng/mL TNF-*α* (Peprotech, Hamburg, Germany) in the serum-starved medium and incubated at 37°C in a humidified atmosphere with 5% CO_2_ and 95% air for 24 hours. As unstimulated control cells were treated equally without addition of TNF-*α* and incubated for the same time period. At the end of the experiment cell culture supernatants were collected and stored at −80°C. Cells were washed once with PBS and lysed immediately in RLT-Buffer (Qiagen, Hilden, Germany) containing 1% mercaptoethanol and stored at −80°C until total RNA isolation. Stimulation experiments were carried out with *n* = 6 wells per experiment for all patients and repeated at least twice for each patient.

### 2.5. RNA Isolation, cDNA Transcription, and Real-Time PCR

Total RNA was isolated from cells using RNeasy kit (Qiagen, Hilden, Germany) according to the manufacturer's instructions. Briefly, after cell lysis with RLT-Buffer containing 1% mercaptoethanol equal volume of 70% ethanol was added and transferred to the RNeasy columns. Columns were washed once with RW1-Buffer and DNA was digested directly at the column using RNase free DNase I (Qiagen, Hilden, Germany) for 15 minutes to avoid genomic DNA contamination. Subsequently, columns were washed again with RW1-Buffer followed by two washing steps with RPE-Buffer. Finally, the total RNA was eluted using water. The yield of purified total RNA was determined by measuring the UV absorbance at 260 nm on the Nanodrop ND1000 spectrophotometer. cDNA was obtained from 250 ng total RNA using the high-capacity kit (Applied Biosystems) according to the manufacturer's protocol and finally diluted to a concentration of 1.25 ng/*μ*L for further analysis. To assess the mRNA expression of the target genes real-time PCR was performed using 5 *μ*L of the gene expression master mix (Applied Biosystems) and the 0.5 *μ*L of the gene expression assay for Col1A1 (Hs00164004_m1), MMP1 (Hs00233958_m1), MMP2 (Hs00234422_m1), MMP3 (Hs00968305_m1), MMP7 (Hs01042795_m1), MMP8 (Hs00233972_m1), MMP9 (Hs00234579_m1), MMP13 (Hs00122992_m1), and MMP14 (Hs00237119_m1) (each includes forward and reverse primers as well the fluorescently FAM-labelled probe) from Applied Biosystems and 1 *μ*L of cDNA in a final volume of 10 *μ*L. Quantification of the house-keeping gene CDKN1b (Hs00153277_m1) as an internal control was performed for each sample. All data were normalized to CDKN1b mRNA level as an endogenous control (unaffected by TNF-*α* treatment). In figures as well as in Table 1 the data are expressed as absolute mRNA expression levels using the formula 2^−ΔCt^. To highlight the different response to external stimuli the data in Table 2 are expressed as relative mRNA expression levels using the formula 2^−ΔΔCt^.

### 2.6. Zymography

The gelatinolytic activity of the cell culture supernatant was determined by gelatine zymography. The samples were mixed with 5x sample buffer and loaded on a 10% sodium dodecylsulfate-polyacrylamide electrophoresis gel copolymerized with 1 mg/mL gelatine. After electrophoresis the gel was incubated in 2.5% Triton X-100 for 60 minutes. The gel was then incubated in 0.05 M TrisHCl, 0.2 M NaCl, 7 mM CaCl_2_, 50 mM MgCl_2_, and 0.2% Brji at 37°C over night. The gel was stained with 0.5% coomassie blue G250 for 3 hours. After destaining the MMP9 activity was detected as clear bands against the blue background. The area of the clear bands was determined using ImageJ and expressed as x-fold over untreated control.

### 2.7. Statistical Analysis

Data are shown as mean ± SEM. For comparison the nonparametric Mann-Whitney *U* test was used. Differences were considered significant when the probability value *P* is lower than 0.05. All analyses were performed using Graph Pad Prism 5.0 software (GraphPad Software, La Jolla, CA).

## 3. Results

### 3.1. Characterization of Human Fibroblasts

**Figure 1:**
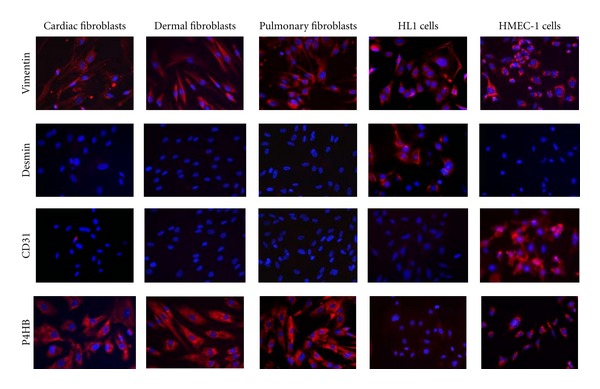
Characterization of primary human fibroblasts. Cells were obtained by outgrowth form tissue samples from heart, skin, and lung. They were stained with antibody directed against vimentin, desmin, CD31, and P4HB. They show positive staining for vimentin and P4HB and were therefore characterized as fibroblasts.

To positively identify the cells populating our primary cultures as fibroblasts, the cells obtained by the outgrowth from biopsies were stained with different markers (Figure 1). Cardiac, dermal, and pulmonary presumable fibroblasts as well as the cardiomyocyte cell line HL1 and the endothelial cell line HMEC-1 expressed the cytosolic localized structural protein vimentin. Furthermore, the primary cells did not express the muscle marker desmin and the endothelial cell surface marker CD31 in contrast to the HL1 and the HMEC-1 cells, respectively. However, the primary cells did exhibit a strongly detectable signal for prolyl 4-hydroxylase (P4HB). This enzyme is essential for the synthesis of collagen, one of the most prominent products of fibroblasts. Consequently, theses cultures contained almost exclusively primary cardiac, dermal, or pulmonary fibroblasts. 

### 3.2. Proliferation

**Figure 2:**
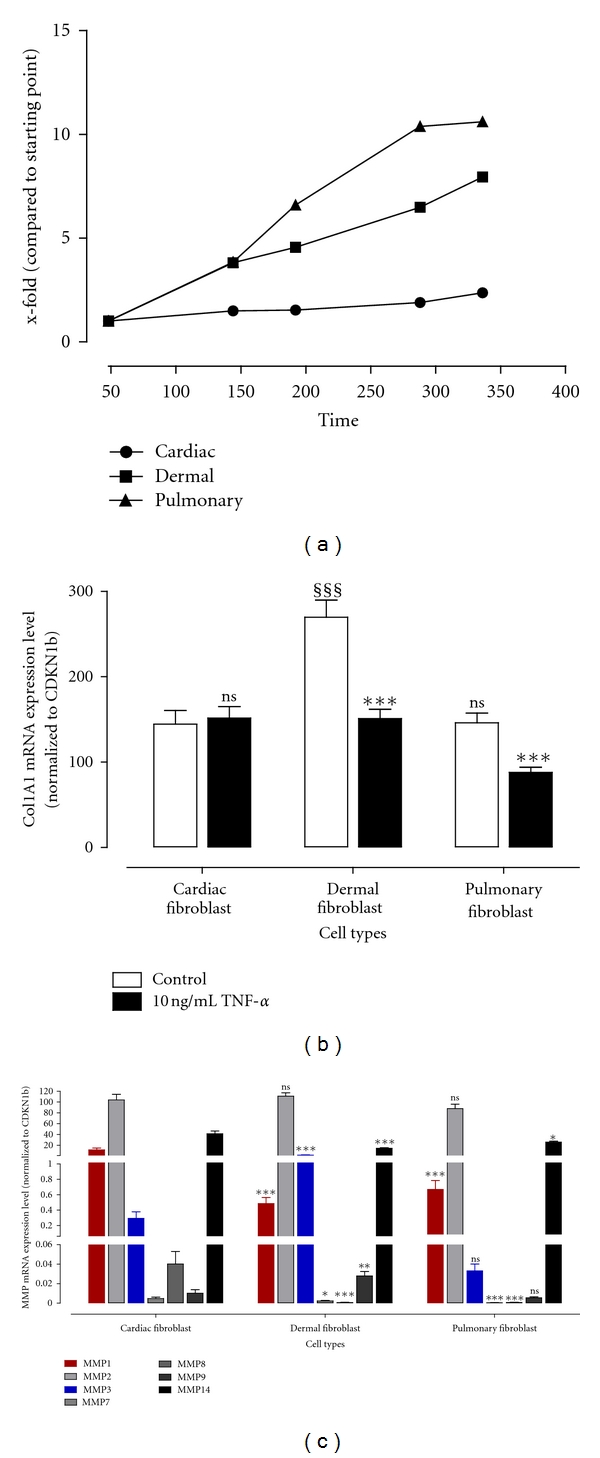
Proliferation, collagen expression, and basal MMP expression of cardiac, dermal, and pulmonary fibroblasts. (a) Growth curves were determined in complete growth medium for fibroblasts derived from heart, skin, and lung. Cardiac fibroblasts reveal a lower proliferation rate compared to dermal and pulmonary fibroblasts. (b) Collagen mRNA expression level was determined using TaqMan from fibroblasts incubated with or without 10 ng/mL TNF-*α* for 24 hours. Untreated dermal fibroblasts show a significantly higher expression level of collagen (^§§§^) compared to untreated cardiac and pulmonary fibroblasts, whereas only in dermal and pulmonary fibroblasts TNF-*α* treatment resulted in significantly decreased collagen expression compared to untreated controls (***). (c) Basal expression levels of different MMPs in dermal and pulmonary fibroblasts were compared to their expression in cardiac fibroblasts. The most obvious difference is shown for MMP1 and MMP3. All mRNA expression levels are shown as absolute expression using the formula 2^−ΔCt^.

To determine the different growth rate of primary human fibroblasts with different origins, cardiac, dermal, and pulmonary fibroblasts were seeded out into 96-well plates and cultured in complete growth medium for 14 days. After different time periods cells were incubated with cellTiter viability assay and the colour development was monitored. The first measurement was done 48 hours after seeding out. Growth curves of cardiac, dermal, and pulmonary fibroblasts in complete growth medium are presented in Figure 2(a). It is clearly shown that cardiac fibroblasts proliferate much slower compared to fibroblasts derived from skin or lung. The calculated doubling time of cardiac fibroblasts was with 12.2 days approximately 4-fold longer compared to the doubling time of 3.7 and 3.3 days of the dermal and pulmonary fibroblasts, respectively.

### 3.3. Basal Expression of Collagen and Matrix Metalloproteases in Cardiac, Dermal, and Pulmonary Fibroblasts

To investigate the basal expression levels of fibroblasts according to their site of origin the mRNA expression level of the untreated control cells after incubation in serum-starved medium for approximately 16 and additional 24 hours was determined using real-time PCR analysis. Therefore, total RNA was extracted from cells and transcribed into cDNA followed by TaqMan for quantification. The determined mRNA expression levels for Col1A1 and MMPs are shown in Figures 2(b) and 2(c), respectively.

A similar collagen 1A1 expression level was found in cardiac and pulmonary fibroblasts. Interestingly an approximately 2-fold higher expression of collagen 1A1 was determined in dermal fibroblasts.

The most abundant MMP in all three types of fibroblasts is MMP2 followed by MMP14. As shown in Table 1 the absolute mRNA expression level of MMP2 does not differ significantly between cardiac, dermal, and pulmonary fibroblasts, whereas the membrane-bound MMP14 (MT1-MMP) is significantly 2.8-fold lower expressed in dermal fibroblasts compared to cardiac fibroblasts.

In all three fibroblast types MMP1 is the most expressed interstitial collagenase whereas the expression in cardiac fibroblasts is significantly 24-fold and 17-fold higher as in dermal or in pulmonary fibroblasts, respectively. The rarely expressed collagenase MMP8 is significantly 63-fold and 54-fold higher expressed in cardiac fibroblasts as well compared to fibroblasts derived from skin or lung, respectively. The collagenase MMP13 could not be detected in all three types of fibroblasts (data not shown).

We further investigated the stromelysins MMP3 and MMP7. Both MMPs are quite low expressed in cardiac fibroblasts, whereas MMP3 is more expressed than MMP7 in all three fibroblast types. When compared to the expression level in cardiac fibroblasts MMP3 is significantly 7.0-fold higher expressed in dermal fibroblasts, whereas it is 8.9-fold lower expressed in pulmonary fibroblasts but in this case it did not reach a significant level (*P* value > 0.05). The stromelysin MMP7 is significantly 2.0-fold and 9.2-fold lower expressed in dermal and pulmonary fibroblasts regarding cardiac fibroblasts, respectively.

The gelatinase MMP9 is one of the lower expressed MMPs in fibroblasts. In dermal fibroblasts the mRNA expression level is 2.7-fold higher and in pulmonary fibroblasts 1.9-fold lower concerning the mRNA expression in cardiac fibroblasts.

### 3.4. Expression of Collagen and Matrix Metalloproteases in Cardiac, Dermal, and Pulmonary Fibroblasts after TNF-*α* Treatment

To explore the influence of TNF-*α* treatment on the expression of collagen we incubated fibroblasts with 10 ng/mL TNF-*α* for 24 hours. After extracting the total RNA and transcribing that into cDNA TaqMan analysis was performed. In Figure 2(b) the impact of TNF-*α* on collagen expression is presented. Whereas TNF-*α* treatment resulted in a 2-fold decreased expression of collagen in fibroblasts derived from skin or lung, there was no influence detectable in cardiac fibroblasts.

Concerning the mRNA expression of MMPs TNF-*α* treatment induced an increased expression of all investigated MMPs in general, whereas the impact of TNF-*α* on the expression of the gelatinase MMP2 and membrane-bound MMP14 (MT1-MMP) did not even reach 2.0-fold increase (Table 2).

**Figure 3:**
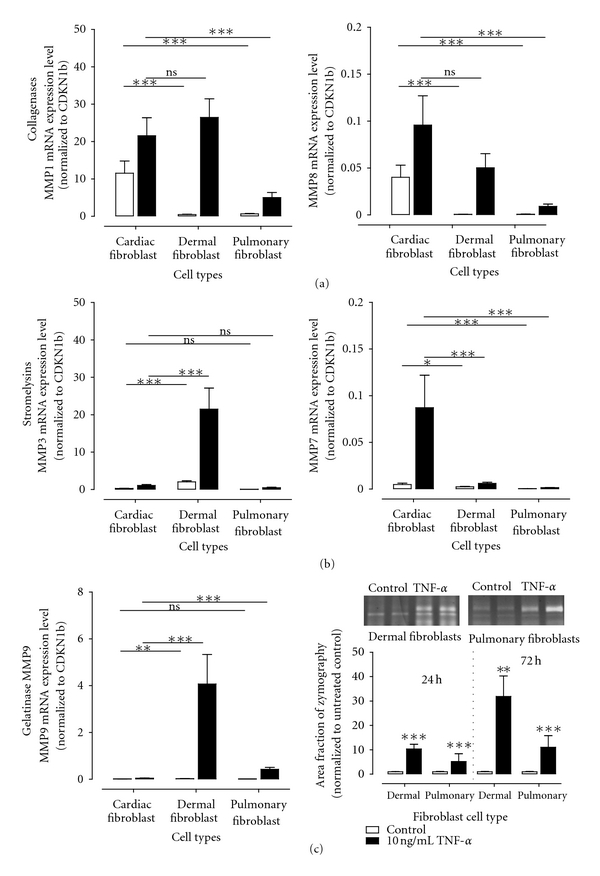
Alteration of MMP expression levels in cardiac, dermal, and pulmonary fibroblasts after treatment with 10 ng/mL TNF-*α* for 24 hours. Different alteration of collagenases (a), stromelysins (b), and MMP9 (c) expression levels after TNF-*α* treatment. MMP9 activity was further determined in the cell culture supernatant of cardiac, dermal, and pulmonary fibroblasts. Representative bands of MMP9 activity in dermal and pulmonary cell culture supernatants with or without TNF-*α* for 72 hours are shown. No MMP9 activity could be detected in cell culture supernatant of cardiac fibroblasts. All mRNA expression levels are shown as absolute expression using the formula 2^−ΔCt^.

In more detail, MMP1 expression was increased 2.0-fold, 100-fold, and 6.0-fold in cardiac, dermal, and pulmonary fibroblasts, respectively (Table 2). In Figure 3(a) it is clearly shown that the TNF-*α* treatment finally leads to a similar mRNA expression of MMP1 in cardiac and dermal fibroblasts, whereas MMP1 is still significantly lower expressed after TNF-*α* treatment in pulmonary fibroblasts compared to the cardiac expression levels. Concerning the TNF-*α*-induced change in expression levels of the collagenase MMP8 similar results could be observed. TNF-*α* leads to an increase of 5.7-fold and 130-fold in cardiac and dermal fibroblasts, respectively, resulting finally in comparable expression levels of MMP8 in cardiac and dermal fibroblasts which demonstrate that no significant difference could be observed after TNF-*α* treatment in contrast to the properties prior to stimulation. In pulmonary fibroblasts the expression of MMP8 was increased by a factor of 10, but still significantly lower than in cardiac fibroblasts.

In cardiac fibroblasts the stromelysin MMP3 expression was 6.7-fold increased by TNF-*α*, whereas in dermal and pulmonary fibroblasts an extended increase of mRNA expression with 9.8-fold and 16-fold was observed, respectively. However, the resulted MMP3 expression did not differ between fibroblasts derived from heart or lung (Figure 3(b)). In contrast TNF-*α* leads with 3.3-fold and 3.1-fold to a slight increase of MMP7 mRNA expression in dermal and pulmonary fibroblasts, respectively. However, in cardiac fibroblasts MMP7 is 16-fold higher expression after TNF-*α* treatment.

Interestingly, a high impact of TNF-*α* on MMP9 expression was observed in dermal and pulmonary fibroblasts (127-fold and 90-fold) whereas in cardiac fibroblasts the resulted change of mRNA expression level was relatively low with an increase of 7.5-fold. Furthermore, to examine the MMP9 secretion zymography was used to determine the protein content of MMP9 in the cell culture supernatants of cardiac, dermal and pulmonary fibroblasts. In the cell culture supernatant of cardiac fibroblasts no MMP9 was detected. In contrast, dermal, and pulmonary fibroblasts secreted low amounts of MMP9 which were significantly increased by TNF-*α* treatment (Figure 3(c)).

## 4. Discussion

In this study we investigated human primary fibroblasts derived from different types of tissue. We examined the proliferation rate, the expression of collagen, and the collagen degrading MMPs of quiescent fibroblasts and compared this basal expression pattern between the different types of fibroblasts. Furthermore, we determined the changes of mRNA expression in these investigated types of fibroblasts after stimulation with the proinflammatory cytokine TNF-*α* to determine differences between fibroblasts isolated from heart, skin, and lung tissues. Interestingly, these three types of fibroblasts reveal major differences in proliferation rate and MMP expression.

Regarding other studies focused on fibroblasts expression we suggested that heterogeneities can be identified [14–17]. It has been reported that even within the single tissue skin fibroblasts occur with different properties concerning the site of origin since dermal fibroblasts from different anatomical sites show similar morphology but their own gene expression profile [17]. Here we focused on the expression pattern of the ECM degradation system which includes the MMPs and compared this between fibroblasts derived from different organs.

First, we characterized these outgrowth cells using fluorescence microscopy. The cultured cells from heart, skin, and lung tissue showed similar morphology. They appear as elongated spindle-shaped cells and revealed positive staining for vimentin and P4HB. To exclude contaminations with other cells than the expected fibroblasts, for instance, endothelial cells or muscle cells such as cardiomyocytes from the heart [18], we stained the cultured cells further with the muscle marker desmin and the endothelial marker CD31. Since nearly no positive cells were obtained by this staining we conclude that the presumable fibroblast cultures from heart, skin, and lung contain almost exclusively primary fibroblasts.

The fibroblast cell type itself is already known as a proliferating cell [3, 19, 20]. Moreover, we compared the growth rate of these fibroblasts according to their site of origin. We clearly demonstrated that fibroblasts derived from skin or lung revealed a much higher proliferation rate compared to cardiac fibroblasts. According to the function of the organ from which the fibroblasts are derived the found differences are explainable. Skin is the largest organ of the body and plays crucial role in protecting against external injuries. Skin wound can arise from environmental influences such as mechanical exposures, surgical procedures, or burns. Most skin wounds can heal naturally due to the existence of highly proliferating cells which could be a satisfying explanation for the determined high growth rate of dermal fibroblasts. An equal proliferation was observed for pulmonary fibroblasts. Again environmental influences are able to reach lung tissue easily by the airway. Therefore, the ability to renew lung tissue is essential as well [10].

In contrast to skin and lung, for cardiac fibroblasts a 4-fold higher doubling time was determined. The heart is not directly open for environmental influences but is situated within the body and only exposed to the closed system the blood circulation. Therefore, outside influences such as bacteria or virus cannot easily reach the heart without passing blood vessels. However, further injuries such as myocardial infarction can occur without direct contact to the environment. But the probability for such injuries of the heart is much lower than for injuries of skin or lung surface. Even if the fibroblast is the most numerous cell type in the heart, cardiomyocytes occupy two-thirds of the tissue volume [7–9]. In contrast to fibroblasts these cells are not able to proliferate after birth [21]. Consequently after myocardial infarction cardiomyocyte death occur, followed by migration and proliferation of fibroblasts and changes in synthesis and degradation of ECM [3].

Since fibroblasts are the common producers of ECM consisting mainly of collagen, we determined the mRNA expression level of the quiescent dermal, pulmonary, and cardiac fibroblasts. Cardiac and pulmonary fibroblasts exhibit an equal expression level of collagen type I in contrast to dermal fibroblasts revealing a 2-fold higher expression. We further investigated the collagen expression of the fibroblast types after TNF-*α* treatment. TNF-*α* is one of the most prominent proinflammatory cytokines which is highly released in response to a large number of inflammatory stimuli, such as LPS or virus antigens [22]. In kidney mesangial cell cultures treated with TNF-*α*, alone a mild but inconsistent reduction in baseline collagen type I level has been detected [23]. Furthermore, TNF-*α* was a consistent and potent inhibitor of the collagen augmentation stimulated by the profibrotic growth factor TGF-*β*. It has been reported that TNF-*α* exerted a suppressive effect on Smad3/4 DNA-binding activity [24] being a potential explanation for the often described antifibrotic effect of TNF-*α* [23–29]. In agreement with these data we found significantly reduced collagen mRNA levels after TNF-*α* treatment in fibroblasts derived from skin and lung but no influence of TNF-*α* on the collagen expression was observed in cardiac fibroblasts.

Next, we focused on the expression of the proteolytic ECM degradation system in quiescent dermal, pulmonary, and cardiac fibroblasts. The MMPs can exist as either soluble or membrane-type MMPs. Soluble MMPs are synthesized in a latent form called pro-MMP. These inactive proenzymes are secreted into the extracellular space where they bind to specific ECM protein and remain quiescent until the prodomain is cleaved off [5]. It is known that this cleaving can be performed by other MMPs [4]. Conversely, the MT-MMPs incorporate a transmembrane region and are inserted into the cell membranes in a fully active state. The family of these enzymes includes a number of members with various and partly overlapping specificities [2, 5]. Therefore, the MMP family is divided into three groups: collagenases (MMP1, MMP8, and MMP13) which predominantly degrade interstitial collagen type I, II, and III, gelatinases (MMP2 and MMP9) degrading gelatins and basements membrane collagen such as collagen IV as well as stromelysins (MMP3 and MMP7) which digest a broad range of substrates such as collagen IV, proteoglycans, laminin, gelatins, and fibronectin [2, 4, 5].

MMP1 is the most expressed soluble collagenase in all three types of fibroblasts, whereas its expression was about 20-fold higher in cardiac fibroblasts compared to dermal and pulmonary fibroblasts. MMP8 is very low expressed but also most expressed in the cardiac fibroblasts. MMP13 could not be detected in all three human types of fibroblasts, whereas this collagenase has been referred to as rodent collagenase, since this is the primary collagenase found in rodents [4].

Another obvious difference in the basal expression pattern of MMPs was found for the stromelysin MMP3 which is most expressed in dermal fibroblasts. It has been reported from fetal skin in comparison to fetal lung fibroblasts that the net-like collagen type IV is relatively high expressed in skin [17]. Since MMP3 is able to digest collagen type IV its higher abundance in skin could be expected.

MMPs catalyze the degradation of ECM macromolecules such as the interstitial and basement membrane collagens and therefore facilitate cell migration of inflammatory cells into the injured tissue. The invading inflammatory cells secrete the proinflammatory cytokine TNF-*α*. Thus we further examined the consequences for MMP expression of fibroblasts after TNF-*α* treatment. It has recently been shown that the proinflammatory cytokine TNF-*α* is one transcriptional activator for MMP9 in dermal human fibroblasts [30].

Collagen type I is the most expressed collagen in the heart. Further collagen I and V are strongly expressed in fetal skin but not in fetal lung fibroblasts [17]. It has already been reported that TNF-*α* induced MMP1 mRNA and protein expression in dermal, gingival, and synovial fibroblasts [31]. In our experiments the TNF-*α* treatment also revealed an extreme increase of the collagenase MMP1 in fibroblasts derived from skin which then reach the equal expression level as determined in cardiac fibroblasts. In almost the same manner in dermal fibroblasts MMP8 was as well increased to a similar level compared to cardiac fibroblasts after TNF-*α* treatment. Finally both soluble collagenases are significantly lower expressed in quiescent dermal and pulmonary fibroblasts but TNF-*α* treatment leads to an equal level of MMP1 and MMP8 as described for cardiac fibroblast in dermal but not in pulmonary fibroblasts.

The expression of the stromelysins MMP3 and MMP7 is as well differentially regulated by TNF-*α*. Whereas MMP3 is most expressed in dermal fibroblasts the expression between cardiac and pulmonary fibroblasts did not differ significantly with or without TNF-*α* treatment. In contrast MMP7 is most expressed and highly up-regulated by TNF-*α* in cardiac fibroblasts compared to fibroblasts from skin or lung.

Furthermore, an extreme upregulation of the gelatinase MMP9 was determined in dermal and pulmonary fibroblasts compared to a weak upregulation in cardiac fibroblasts. This difference leads to detectable secreted MMP9 protein using zymography in dermal and pulmonary fibroblasts but not in cardiac fibroblasts. One substrate of MMP9 is collagen IV, the net-like collagen which forms the basement membrane, and therefore MMP9 facilitates cell migration into the injured tissue.

As already mentioned, the activity of MMPs is regulated through transcriptional activators such as TNF-*α*. Furthermore the presence of active MMPs is also regulated by control of posttranslational cleaving and the presence of endogenous inhibitors so-called tissue inhibitor of MMPs (TIMPs). These TIMPs bind noncovalently to the catalytic domain of MMPs in a 1 : 1 stoichiometry and therefore prevent the access of the substrate to the active site of the enzyme [32]. Proteolysis becomes pathological, when the normal balance between MMPs and their inhibitors TIMPs is lost. In this study we focused on the transcriptional regulation but several studies have been performed to get a better understanding for the posttranslational control. For instance in the presence of MMP3 a 12-fold increase, in the conversion of pro-MMP1 to active MMP1 was reported [33]. In other studies, inflammatory cytokines such as IL-1*α* are known to play a major role in regulating MMP and TIMP expression in several inflammatory diseases. The stimulatory effect of IL-1*α* as well as TNF-*α* has been reported for both the expression of MMPs and TIMPs in dental pulp fibroblasts which leads to an alteration of the balance between the MMPs and TIMPs [34, 35].

Beyond their effects on matrix degradation to facilitate the invasion of inflammatory cells, MMPs can further modulate inflammatory pathways by processing cytokines, chemokines, and growth factors. For example, MMP7 and the membrane-bound MMP14 have both been identified to process membrane-bound TNF-*α* to a soluble active TNF-*α* form enhancing its biological activity [36]. On the other hand MMPs can cleave chemokines which are responsible for recruiting more inflammatory cells to the site of injury. Chemokine cleavage by MMPs most often results in reduced chemokine activity [37–39]. It has been especially shown for CC-chemokines that MMP cleavage creates chemokine receptor antagonists and thus MMPs damp inflammation and are strongly involved in a kind of negative feedback mechanism to prevent too extensive inflammation [39] (Westermann et al. 2011, accepted) [40]. 

In the past the fibroblasts have typically been understood as a uniform cell population with comparable function regardless of their origin. Here we demonstrated serious differences in proliferation, collagen synthesis as well as MMP expression under basal or inflammatory conditions. However, an excess of MMPs resulting in degradation of ECM is thought to be a key feature of the pathology of various inflammatory and degenerative diseases [41–43]. Since extensive heterogeneity among fibroblasts from different tissues has been demonstrated, it is essential to examine cardiac fibroblasts to understand myocardial dysfunctions even if it is more difficult to obtain and to cultivate cardiac fibroblasts.

## Figures and Tables

**Table 1:** Absolute mRNA expression levels of different MMPs in cardiac, dermal, and pulmonary human fibroblasts. The mRNA expression was normalized to the housekeeping gene CDKN1b using the formula 2^−Δ*Ct*^. For statistical significance dermal or pulmonary expression was compared to the expression in cardiac fibroblasts using Mann-Whitney *U* test.

	Absolute mRNA expression level						
	(normalized to CDKN1b)						
Gene	Cardiac fibroblast	Dermal fibroblast			Pulmonary fibroblast		
	Expression	Expression	x-fold to cardiac fibroblast	*P* value	Expression	x-fold to cardiac fibroblast	*P* value
MMP1	12 ± 3	0.49 ± 0.08	**−24**	<0.0001	0.67 ± 0.12	**−17**	<0.0001
MMP2	104 ± 10	111 ± 6	**1.1**	0.559	88 ± 8	**−1.2**	0.411
MMP3	0.29 ± 0.08	2.1 ± 0.3	**7.0**	<0.0001	0.033 ± 0.007	**−8.9**	0.155
MMP7	0.005 ± 0.001	0.0024 ± 0.0006	**−2.0**	0.013	0.0005 ± 0.0001	**−9.2**	<0.0001
MMP8	0.040 ± 0.013	0.0006 ± 0.0002	**−63**	<0.0001	0.0007 ± 0.0002	**−54**	<0.0001
MMP9	0.010 ± 0.003	0.028 ± 0.005	**2.7**	0.003	0.0055 ± 0.0012	**−1.9**	0.292
MMP14	41 ± 5	15 ± 1	**−2.8**	<0.0001	26 ± 2	**−1.6**	0.033

**Table 2:** Relative mRNA expression level of different MMPs in cardiac, dermal, and pulmonary human fibroblasts after treatment with 10 ng/mL TNF-*α* for 24 hours. The mRNA expression was normalized to the housekeeping gene CDKN1b and to the according untreated control using the formula 2^−ΔΔ*Ct*^ and is given as x-fold increase. For statistical significance treated and untreated control were compared using Mann-Whitney *U* test.

	relative mRNA expression level after TNF-*α* stimulation for 24 hours					
	(normalized to unstimulated control)					
Gene	Cardiac fibroblast		Dermal fibroblast		Pulmonary fibroblast	
	expression	*P* value	expression	*P* value	expression	*P* value
	(x-fold to control)		(x-fold to control)		(x-fold to control)	
MMP1	2.0 ± 0.2	0.044	100 ± 12	<0.0001	6.0 ± 0.8	<0.0001
MMP2	1.6 ± 0.1	0.0002	1.2 ± 0.1	0.023	1.2 ± 0.0	0.007
MMP3	6.7 ± 1.2	<0.0001	9.8 ± 1.8	<0.0001	16 ± 3	<0.0001
MMP7	16 ± 4	<0.0001	3.3 ± 0.3	<0.0001	3.1 ± 0.5	0.0006
MMP8	5.7 ± 1.2	<0.0001	130 ± 50	<0.0001	10 ± 2	<0.0001
MMP9	7.5 ± 2.4	0.005	127 ± 27	<0.0001	90 ± 11	<0.0001
MMP14	1.6 ± 0.1	0.018	1.6 ± 0.3	0.103	1.2 ± 0.1	0.317
